# Band Gap Reduction in Ferroelectric BaTiO_3_ Through Heterovalent Cu-Te Co-Doping for Visible-Light Photocatalysis

**DOI:** 10.3389/fchem.2021.682979

**Published:** 2021-05-24

**Authors:** Rohit Kumar Rohj, Akmal Hossain, Priya Mahadevan, D. D. Sarma

**Affiliations:** ^1^ Solid State and Structural Chemistry Unit, Indian Institute of Science, Bengaluru, India; ^2^ S. N. Bose National Centre for Basic Sciences, Kolkata, India

**Keywords:** photocatalysis, co-doping, ferroelectrics, DFT, Jahn-Teller distortions, work function

## Abstract

It is believed that ferroelectrics may serve as efficient photocatalysts as well as photovoltaic materials but for their large bandgaps which does not allow them to absorb a large part of the solar spectrum. We have explored theoretically within ab-initio density functional theory-based calculations, the efficacy of Cu and Te to co-dope BaTiO_3_ in reducing its bandgap while retaining its ferroelectric properties. Examining a dopant concentration of 11%, we find an insulating ground state being realized with a band gap reduction of 0.42 eV from the value for undoped BaTiO_3_ for some doping configurations. Ferroelectric distortions are found to survive even in the presence of doping suggesting possible applications in photocatalysis as well as photovoltaics.

## Introduction

An increasing demand for energy has resulted in an over utilization of available natural resources, with most of them being non-renewable fossil fuels. This has led to a rapid increase in carbon emissions, causing global warming and environmental pollution, requiring an urgent need to find ways for sustainable energy production. Among the various ways and techniques to cope with the problem, photocatalysis and photovoltaics utilizing sunlight as a clean, inexpensive, and inexhaustible source are being extensively explored to face these challenges. Photocatalysis, in particular, has been applied in CO_2_ reduction (Zhao et al., 2017), H_2_ evolution via water splitting (Walter et al., 2010), dye degradation (Ajmal et al., 2014) and ammonia synthesis (Chen et al., 2018; Zhao et al., 2019). Since the seminal work of Fujishima and Honda (Fujishima and Honda, 1972) of using TiO_2_ as a photocatalyst in 1972, various semiconducting materials including WO_3_ (Dong et al., 2017), ZnO (Ong et al., 2018), Bi_2_O_3_ (Zhou et al., 2009), Bi_5_O_7_I (Cao et al., 2020), oxide tandem structures (Enesca, 2020) and CdS (Cheng et al., 2018) have been extensively investigated for photocatalytic applications. The operation of a photocatalyst has primarily three parts. The first involves using sunlight to generate electron-hole pairs in the material. The second part involves separating the electron and hole pairs before they recombine. Finally, the free electrons and holes need to be transported to the surface for surface redox reactions. Hence, in a material that is optimal for this purpose, the band gap should lie in a range that maximizes absorption of the solar spectrum. This corresponds to a band gap in the range of 1–1.5 eV where the solar spectrum peaks. Additionally, a material with a high absorption coefficient would also help in maximising the photo-generated carriers. The next step involves separating the electron and hole before they recombine. An indirect band gap material or a heterostructure of materials with Type II band alignment would be appropriate for this purpose. And finally, the surface potentials should have values that would be optimal for the envisaged reaction on the surface. To summarize, the important processes involved in the operation of a photocatalyst are charge generation via photo-absorption, charge separation, and charge collection. Out of these, charge collection is an interfacial phenomenon which depends on the energetics of electrons and holes and the medium involved in the particular reaction that one is considering. Since these interfacial effects would be reaction specific, we focus our attention on the present work to the issues of photo-absorption and charge separation.

Ferroelectric materials have a built-in potential due to their intrinsic electric polarization within each domain. This has been found to be useful in separating the electron-hole pairs as they migrate towards the surface before recombination (Stock and Dunn, 2012; Cui et al., 2013; Li et al., 2014; Wang et al., 2017). Most ferroelectrics have large band gaps and are hence unsuitable for this purpose. Optimising the band gap is usually achieved by co-doping with a combination of atoms which helps to retain the insulating, ferroelectric character, while at the same time reducing the band gap. Unfortunately, attempts to reduce the bandgap by doping other elements into a ferroelectric material most often lead to a rapid loss of ferroelectric properties, making the design of a small bandgap ferroelectric remain mostly elusive (Hill, 2000; Benedek and Fennie, 2013). Das et al. (2018), however, showed that a heterovalent, charge-compensated co-doping of Jahn-Teller Mn^3+^ ions and d^0^ Nb^5+^ ions into BaTiO_3_, led to one of the smallest bandgap materials largely retaining ferroelectric properties of BaTiO_3_. This work also showed that the reduced band gap is determined by the Jahn-Teller splitting of the Mn^3+^ states. The dominantly Nb states were empty and did not contribute to the states in the band gap. A final requirement of any material chosen to be a photocatalytic material is that it should be chemically and thermally stable and should be resistant to photodegradation. It is interesting to note that most of the desirable attributes of a good photocatalyst, such as the right bandgap for the absorption of the large part of the solar spectrum, retention of ferroelectric properties for facile charge separation and a high degree of absorption, also make such materials suitable to explore efficient solar photovoltaic applications.

In this work, we theoretically explore the possibility of expanding the strategy introduced by Das et al. (2018) of charge-compensated heterovalent doping of a ferroelectric material with a Jahn-Teller active ion and a closed shell metal ion to achieve a lower bandgap compound that retains ferroelectric properties. We have considered the well-known ferroelectric BaTiO_3_. This has been co-doped with Cu and Te at the Ti^4+^ site. The choice has been dictated by the similar ionic radii of the Jahn-Teller active d^9^ Cu^2+^ (0.73 Å) which is the most stable valency of a Cu atom, and Ti^4+^ (0.60 Å) ion. This would lead to a valency of 6+ on the Te sites with an ionic size of 0.56 Å and a closed shell electronic configuration. Our results establish that Cu 3d derived states appear within the bandgap of the host BaTiO_3_. The band gap calculated within generalised gradient approximation for the exchange correlation functional employed here gives us a band gap of the co-doped system to be 1.25 eV while retaining its ferroelectric polarisation to a substantial extent, making this system an exciting one for possible photocatalytic and photovoltaic applications.

## Computational Methodology

We have performed density functional theory (DFT) calculations within a plane wave basis set using projector augmented wave (PAW) potentials as implemented in the Vienna *ab initio* Simulation Package (VASP). The experimental lattice constants of BaTiO_3_ (BTO), **a** = **b** = 3.992 and **c** = 4.033 Å for the room temperature tetragonal phase, were taken and a unit cell was constructed allowing for the internal coordinates to be relaxed. After optimization, the Ti-O bond lengths in the **c**-direction were found to be 1.885 and 2.148 Å. A supercell was constructed out of the optimized unit cell with 3 × 3 × 2 repeat units along the three cartesian directions, and pairs of Ti atoms at various separations were replaced by a Cu and a Te atom to realize different configurations at a fixed level of doping (∼11%). We have optimized the internal positions for each of these co-doped configurations till the forces on each atom were less than 10^−2^ eV/Å. Generalized Gradient Approximation (GGA) has been used for the exchange-correlation functional. An energy of 550 eV was chosen for the maximum kinetic energy of the plane waves included in the basis. A mesh of 6 × 6 × 6 *k*-points was used for the *k*-points integrations involved for the supercell and an appropriately scaled mesh for the primitive unit cell. In addition, beyond GGA calculations were also performed for the doped unit cells by including on-site Coulomb interactions of 6 eV at the Cu site using the Dudarev implementation (Dudarev et al., 1998). Spheres of radii of 1.31 were constructed about the Cu atoms for calculating the atom projected partial density of states as well as the magnetic moment at each atomic site. In order to calculate the work function (Φ), we consider two symmetric slabs containing 13 monolayers of tetragonal ferroelectric BaTiO_3_, one terminating with BaO on both ends and the other one with TiO_2_. 12 Å of vacuum has been introduced in the z-direction in both the slabs and in the xy plane, we have taken a 2 × 2 unit cell and two Ti atoms are replaced by a Cu atom and a Te atom at a distance as in configuration IV. The layer averaged potential is calculated after the self-consistent spin polarised density functional theory calculation for the slabs considered. The difference of the potential in the vacuum region where it is constant, and the fermi energy is used to define the work function (Kumar and Niranjan, 2014).

## Results and Discussion

**FIGURE 1:**
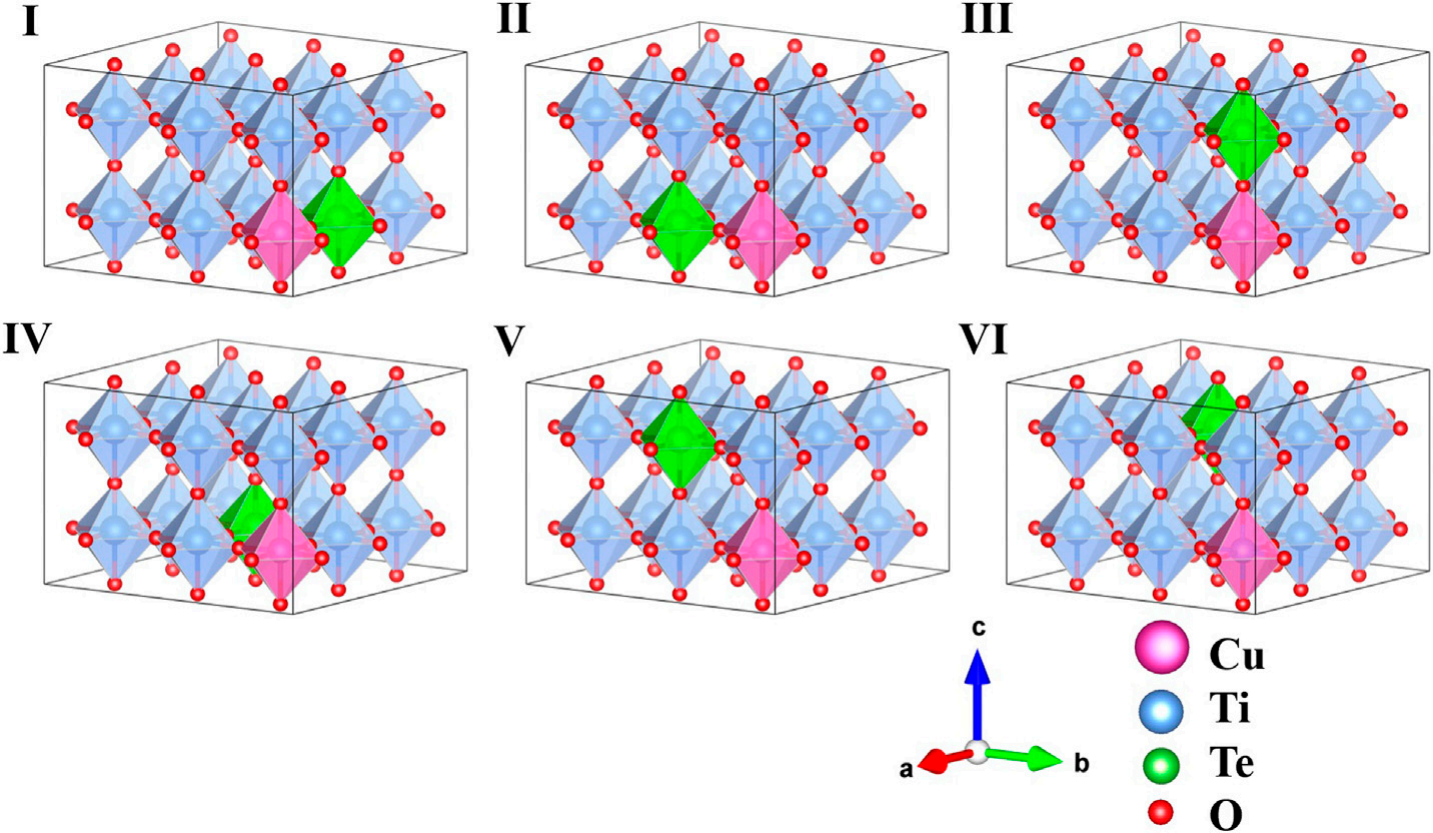
Schematic representation of the six possible symmetry distinct configurations of 3 × 3 × 2 perovskite supercell of Cu-Te co-doped BaTiO_3_. Barium (Ba) atoms are not shown for clarity.

The prescription of co-doping with Cu and Te to replace two Ti ions in the BTO supercell leads to six distinct configurations, labelled I-VI in order of their increased Cu-Te separation, and are shown in Figure 1. The tetragonal symmetry of the lattice makes configurations I and II, in which the dopant atoms sit at nearest neighbor Ti positions along the **a** and **b** lattice vector directions, identical. Configuration III has the dopant atoms lie on top of each other along the **c** axis. The rest of the configurations IV, V and VI correspond to next nearest neighboring geometry where the co-dopant distances are 5.65, 5.67, and 6.94 Å, respectively. The ground states obtained in each case are given in Table 1. All energies have been calculated per formula unit and have been given for each configuration relative to configuration III, since configuration III is found to have the lowest energy.

**TABLE 1:** Energies and metal oxygen bond lengths in the MO_6_ octahedra for geometry optimized DFT calculations for all six configurations of BaTi_1-x_(Cu_1/2_Te_1/2_)_x_O_3_ with x = 0.11. The axes having the largest off centering distortion in Ti-O bond lengths have been highlighted in bold.

Configuration	Relative stability (meV/f.u.)	Bond length (in Å)								
		Axis	^1^Ti-O		^2^Ti-O		Cu-O		Te-O	
I (−690.379 eV)	12	a	**2.042**	**1.942**	**2.067**	**1.932**	**2.106**	**2.147**	**1.891**	**1.904**
		b	1.997	1.997	1.997	1.997	2.072	2.072	1.958	1.958
		c	**1.942**	**2.076**	**1.902**	**2.130**	**2.091**	**2.098**	**1.978**	**1.991**
II (−690.379 eV)	12	a	1.997	1.997	1.997	1.997	2.072	2.072	1.958	1.957
		b	**2.042**	**1.942**	**2.067**	**1.932**	**2.106**	**2.147**	**1.891**	**1.904**
		c	**1.942**	**2.076**	**1.902**	**2.130**	**2.091**	**2.098**	**1.978**	**1.991**
III (−690.596 eV)	0	a	1.994	1.994	1.997	1.997	2.064	2.064	1.977	1.977
		b	1.994	1.994	1.997	1.997	2.064	2.064	1.977	1.977
		c	**1.906**	**2.128**	**1.892**	**2.141**	**2.148**	**2.142**	**1.888**	**1.888**
IV (−690.024 eV)	31	a	1.993	1.993	2.008	1.988	2.115	2.128	1.923	1.925
		b	1.993	1.993	2.008	1.988	2.115	2.128	1.923	1.925
		c	**1.936**	**2.089**	**1.897**	**2.130**	**2.100**	**2.113**	**1.958**	**1.973**
V (−690.072 eV)	29	a	1.998	1.998	1.996	1.996	2.107	2.107	1.933	1.933
		b	1.998	1.980	2.054	1.944	2.120	2.132	1.922	1.922
		c	**1.937**	**2.080**	**1.900**	**2.136**	**2.116**	**2.124**	**1.950**	**1.961**
VI (−689.994 eV)	33	a	2.002	1.987	2.044	1.953	2.099	2.122	1.927	1.930
		b	2.002	1.987	2.044	1.953	2.099	2.122	1.927	1.930
		c	**1.930**	**2.091**	**1.902**	**2.133**	**2.105**	**2.117**	**1.955**	**1.969**

For configurations I and II with the dopant atoms at nearest neighbor positions along the **a** and **b** lattice vectors are substantially higher by 12 meV per formula unit. Energies for configurations with the dopant atoms at further distances are much higher appearing about 29–33 meV above that of configuration III. The metal-oxygen (M-O) bond lengths for Cu and Te in their respective octahedra have been tabulated for all configurations in Table 1. The observed bond lengths seem to be consistent with the assigned valencies of 2+ for Cu and 6+ for Te. Due to the larger ionic radius of Cu^2+^ than Te^6+^, all Cu-O bond lengths are found to be longer than Te-O bond lengths (Longo and Raccah, 1973; Shannon, 1976; Flores et al., 2019).

In an octahedral symmetry environment arising from the ligands surrounding a transition metal site, one has a degeneracy lifting of the d orbitals into triply degenerate t_2g_ orbitals and doubly degenerate e_g_ orbitals. Examining the distortions in configuration III, we find that the two out-of plane Cu-O bond lengths are almost equal to each other, while these are considerably longer than the in-plane Cu-O bond lengths. This shows that the local symmetry is not octahedral but D_4h_, establishing that Jahn-Teller effects are operational, as expected for the 3d^9^ electronic configuration of Cu^2+^. This distortion lifts the degeneracy of the e_g_ orbitals with the d_x_^2^_−y_^2^ orbitals lying at higher energies than the d_3z_^2^_−r_^2^ orbitals. It is to be noted that the local distortion around the Cu^2+^ and Te^6+^ in configuration III are essentially non-polar in contrast to the polar distortions seen at the Ti^4+^ sites in Table 1 for all configurations, responsible for the ferroelectricity. Thus, it appears that the Cu and Te co-doping sustains the ferroelectric distortions at the Ti site while not directly contributing via any ferroelectric distortions of the dopant ions. It is interesting to note that the presence of the dopant pairs in configurations I and II introduces an off-centering in the nearest neighbor Ti sites in the **a** or the **b** direction. This arises from inversion symmetry breaking in the structure, leading to the particular Ti atom having different neighbors on either side. This also induces an off-centering at the Cu sites. It is possibly from these additional lattice distortions than what one finds in configuration III that make configurations I and II have higher energy. Analysing the local geometry around the dopant sites, we have classified two kinds of Ti atoms in these supercell configurations. The ones which are immediate neighbors of dopant atoms are labelled as ^1^Ti and others which are not immediate neighbors are labelled as ^2^Ti as shown in Table 1. The local geometry around the dopant atoms in these relaxed supercells show that both the type of Ti atoms (^1^Ti and ^2^Ti) exhibit significant off-centering distortion as reflected in the dissimilar Ti-O bond lengths (marked in bold) and are averaged over the neighboring TiO_6_ octahedra. This indicates that the off-centering of Ti^4+^ ions survive the doping. This is the most important criterion to make use of the ferroelectric polarization-generated electric field to efficiently separate photo-generated electrons and holes using the internal electric field.

**FIGURE 2:**
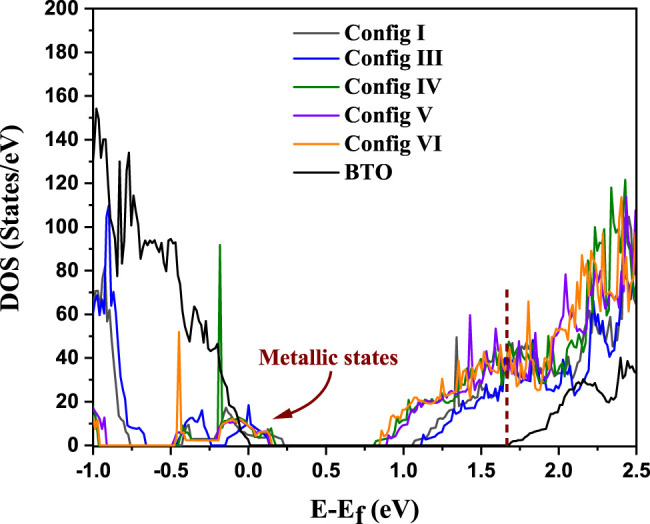
DOS calculated using DFT (U = 0) for all six configurations for BaTi_1-x_(Cu_1/2_Te_1/2_)_x_O_3_ where x = 0.11 and for pure BTO. Dotted line at 1.67 eV represents the conduction band edge for BTO.

It is obvious that any photocatalytic or photovoltaic material must be an insulator with a finite energy gap. In absence of any such gap, the photoexcited electrons and holes will rapidly de-excite to the chemical potential to recombine without giving rise to any free electron and hole for any useful purpose. We examine if the dopant pairs that we have chosen lead to an insulating state. This is examined by calculating the density of states for configurations I to VI. The configurations I and II are essentially the same by tetragonal symmetry, thus we consider only one of them. For comparison, we also show the density of states for undoped BTO. The results are shown in Figure 2. The undoped BTO system is found to have a band gap of 1.67 eV in our calculations in contrast to the experimental value of 3.2 eV(Das et al., 2018). This underestimation of the band gap within GGA calculations is well-known. The insulating state of BTO is demonstrated by the absence of any density of electron states between the chemical potential at the top of the valence band (VB), marked by zero of the energy scale, and the bottom of the conduction band (CB) at 1.67 eV. Upon doping, all the supercell configurations (I–VI) are found to be metallic as shown by the finite density of electron states at the chemical potential. This is to be expected as Cu^2+^ has a partially filled d^9^ configuration. The Jahn-Teller distortions discussed earlier in the context of configuration III will lead to the levels with dominantly d_x_^2^_−y_^2^ character with spin degeneracy but being occupied by one electron, thereby, making the system metallic. Similar arguments can be extended to all other configurations. Since spin-polarization in the Cu 3d states can lift the spin-degeneracy of the d_x_^2^_−y_^2^ orbitals at the Cu site, potentially turning the system into an insulator via spin-splitting of the d-band, it is important to explore spin polarisation in such calculations. It is well-known that the intra-site Coulomb interaction strength, *U*, within the Cu 3d states is large and it can drive the system to an insulating state even in absence of any spin-polarization.

We explore the consequence of Coulomb interactions and spin polarization by carrying out spin-polarized calculations within the GGA + U approach, discussed in the *Computational Methodology* section. This led to a magnetic moment of 0.71 *μ*_*B*_ developing on the Cu site. Following the literature (Isseroff and Carter, 2012), we set *U* to a typical value of 6 eV within Cu 3d states. Among the six analysed configurations, we have selected configuration III, with its lowest energy, serving the purpose of nearest neighbor geometry where dopant atoms separation is equal to the unit cell length in the **c** direction and configuration IV and VI serving as non-nearest neighbor geometries where the dopant atoms separation is 5.65 and 6.94 Å, respectively. Structural optimizations of three configurations were carried out allowing for spin polarizations of the states as well as including *U* = 6 eV within the Cu 3d states. Analysis of the ground state energies and bond lengths for these three chosen configurations are shown in Table 2.

**TABLE 2:** Energies and metal oxygen bond lengths in the MO_6_ octahedra for geometry optimized DFT + U (U = 6) calculations for the three configurations III, IV and VI of BaTi_1-x_(Cu_1/2_Te_1/2_)_x_O_3_ with x = 0.11. The axes having the largest off centering distortion in Ti-O bond lengths have been highlighted in bold.

Configuration	Relative stability (meV/f.u.)	Bond length (in Å)								
		Axis	^1^Ti-O		^2^Ti-O		Cu-O		Te-O	
III (−689.299 eV)	0	a	1.996	1.996	1.998	1.998	2.057	2.057	1.967	1.967
		b	1.996	1.996	1.998	1.998	2.057	2.057	1.967	1.967
		c	**1.900**	**2.134**	**1.891**	**2.142**	**2.146**	**2.148**	**1.884**	**1.888**
IV (−688.522 eV)	43	a	1.999	2.001	2.004	1.989	2.079	2.084	1.925	1.926
		b	1.999	2.001	2.004	1.989	2.079	2.084	1.925	1.926
		c	**1.924**	**2.077**	**1.897**	**2.138**	**2.139**	**2.152**	**1.957**	**1.972**
VI (−688.514 eV)	44	a	1.996	2.004	2.018	1.977	2.061	2.082	1.927	1.930
		b	1.996	2.004	2.018	1.977	2.061	2.082	1.927	1.930
		c	**1.920**	**2.086**	**1.897**	**2.008**	**2.142**	**2.155**	**1.956**	**1.970**

**FIGURE 3:**
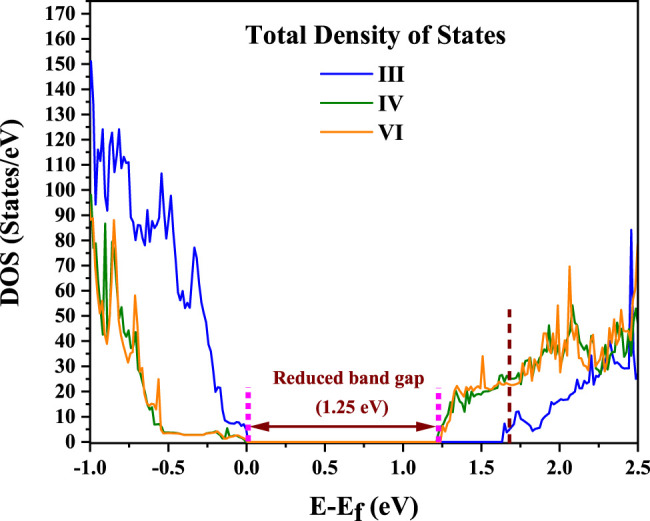
Spin polarized total density of states (TDOS) calculated using DFT + U (U = 6 on Cu) for the configurations III, IV and VI for BaTi_1-x_(Cu_1/2_Te_1/2_)_x_O_3_ where x = 0.11. Vertical dotted line at 1.67 eV shows the CB edge for pure BTO. The zero of energy is the valence band maximum.

**FIGURE 4:**
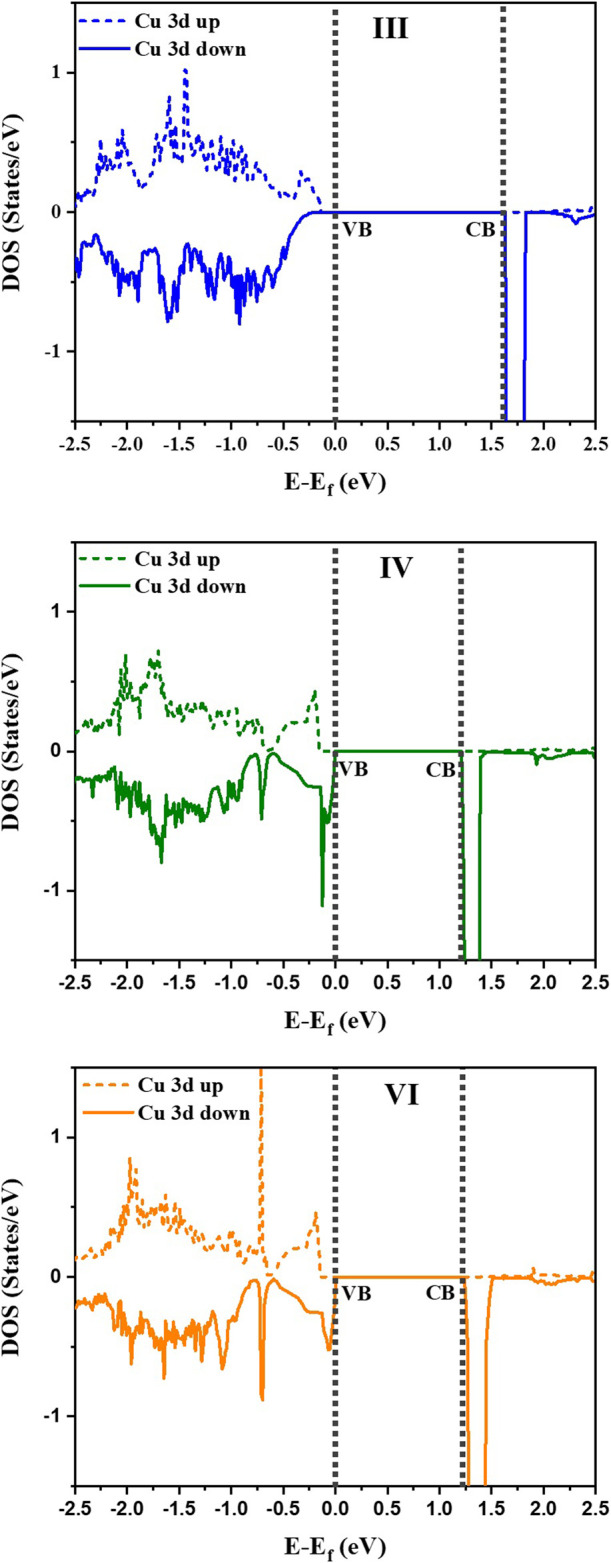
Partial Density of States (PDOS) for Cu 3d spin up and spin down states for the configurations III, IV and VI. Vertical dotted lines represent VB and CB of the three doped configurations. The zero of energy is the valence band maximum.

It is observed that the configuration III has the lowest energy as in the earlier calculations without *U* or spin polarization. Examining the distortions of the Cu-O, Te-O and Ti-O octahedra, we see that the bond lengths turn out to be similar to the earlier result given in Table 1. The off-centering is sustained in the **c**-direction for the Ti atoms, while Cu^2+^ exhibits a strong tetragonal distortion leading to longer Cu-O bonds in the **c**-direction arising from first order Jahn Teller effects. Te^6+^ responds to the changes in the Cu-O bond lengths and has shorter Te-O bond lengths in the **c**-direction but has no second order Jahn-Teller effects operational and consequently has no off-centering of the Te atoms. Other features of the distortions for configurations IV and VI remain similar to what was found in Table 1 with a marginal increase in the stability of configuration III with respect to configurations IV and VI. The most significant change in the electronic structure brought about by the inclusion of the spin polarization together with finite *U* is that all co-doped systems are driven into insulating states with a clear bandgap, as shown in Figure 3. As mentioned already, this is critical for possible applications of any compound as a photocatalyst or a photovoltaic material. Comparison of densities of states for different configurations with that of pure BTO, shown in Figure 3, establishes a significant reduction in the band gap to 1.25 eV for configurations IV and VI as compared to pure BTO which is 1.67 eV shown by the vertical dotted line. The lowest energy configuration III, however, does not exhibit a significant reduction in the band gap. In order to examine the origin of the reduction in the band gap, we examine the Cu 3d projected up and down spin partial density of states. This is plotted in Figure 4 for each configuration. The vertical dashed lines indicate the positions of the valence and conduction band edges in each case. We find that the occupied Cu 3d states lie deep inside the valence band in configuration III with the host BTO states contributing to the valence band top.

The conduction band bottom, however, has the Cu d minority spin states almost merging with the host states indicating that in this case, one has a very small reduction in the band gap by co-doping. Both configurations IV and VI exhibit a significant reduction in the band gap of 0.42 eV. This seems surprising at first sight as the distortions of the Cu-O bond lengths are not significantly different in the three cases. This should therefore lead to similar band gap reductions. A possible origin for this could be the choice of the host supercell which has just two repeat units in the **c** direction. This leads to a chain formation of alternating Cu-Te atoms in the **c** direction for configuration III leading to the positioning of the unoccupied minority spin Cu d states just at the bottom of the conduction band. As this is a special consequence of the choice of unit cell, dictated by the increased computational demands for considering three repeat units in the **c** direction, we can safely conclude that the band gap reduction in the three cases should be similar.

**FIGURE 5:**
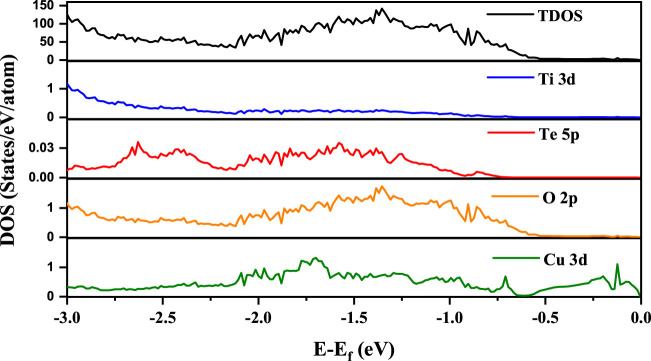
PDOS for Ti 3d, Te 5p, O 2p and Cu 3d for the studied configuration IV in addition to the total density of states. The zero of energy is the valence band maximum in our calculations.

Having established that by co-doping Cu and Te in BTO we are able to realize an insulating ground state with a reduced gap in which ferroelectric distortions of the Ti atoms still survive, we show the partial density of states (PDOS) in Figure 5 for Ti 3d, Te 5p, O 2p and Cu 3d along with the total density of states (TDOS) in the energy interval from the top of the valence band to 3 eV below for one of the three configurations (config IV here) that we have studied.

**FIGURE 6:**
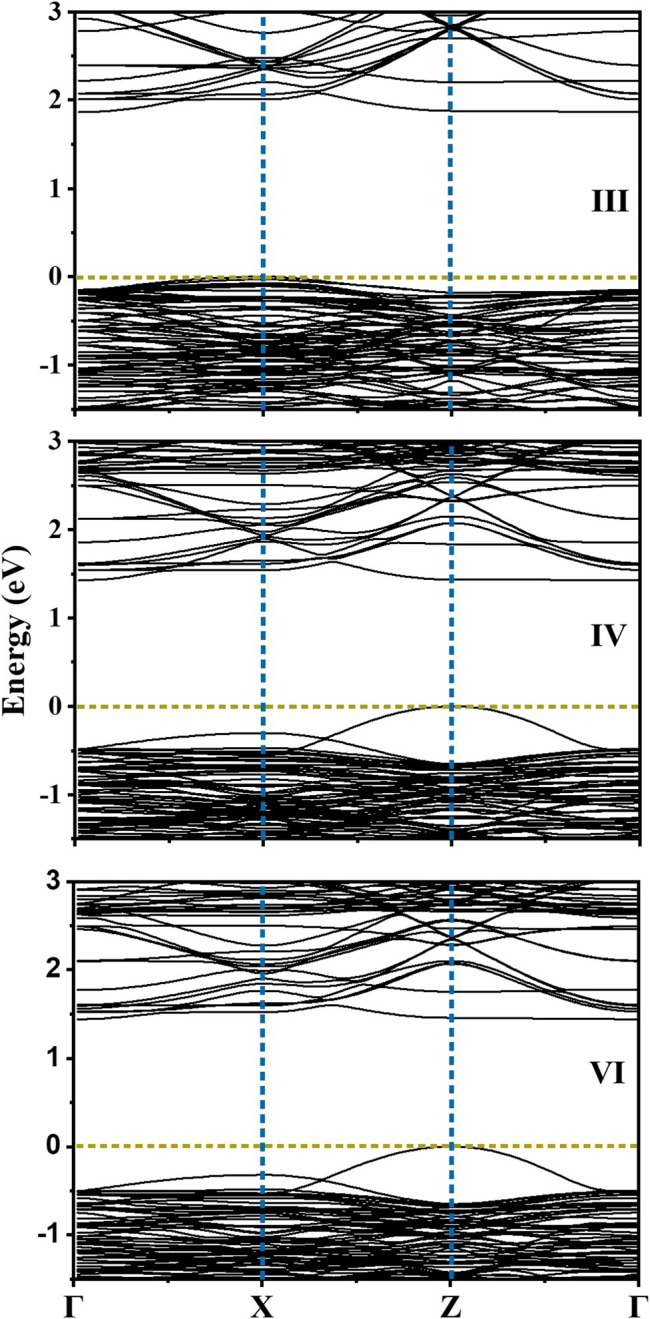
Electronic band structure calculated for the configurations III, IV and VI for BaTi_1-x_(Cu_1/2_Te_1/2_)_x_O_3_ where x = 0.11 along the high symmetry *k*-points.

**FIGURE 7:**
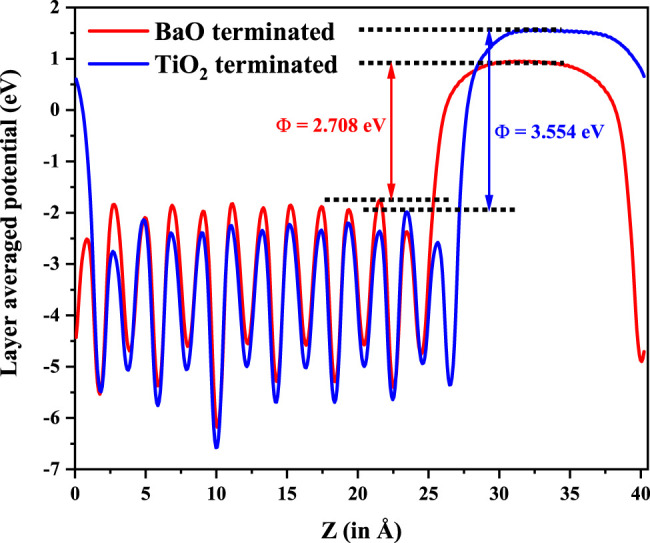
Work function evaluated for BaO and TiO_2_ termination at both end surfaces for 2 × 2 × 6.5 supercell of BaTiO_3_ with Cu and Te co-doping.

From the position of the Cu d states in the valence band, it is evident that the Cu d states occur in the band gap of BaTiO_3_. Their position can be experimentally probed by measuring the ultraviolet photoemission spectrum (UPS). The given partial density of states weighted by the cross-section at the particular photon energy used, may be used to compute the UPS. It is clearly seen that valence band spectrum in TDOS is mainly contributed by O 2p states. We then go on to examine the nature of the band gap. This is plotted along various symmetry directions in Figure 6 for each configuration. In every case, the valence band top is contributed by a strongly dispersing band with Cu d character. This was evident from our analysis of the density of states also. The conduction band bottom is contributed by Cu d_x_^2^_−y_^2^ states which are weakly dispersive in the ГZ direction, with the minimum lying at Г point. As the band extrema corresponding to the valence band maximum and the conduction band minimum are at different k-points, leading to an indirect bandgap, one expects the recombination rates to be low, which again is another requirement for a material to work as an efficient photocatalyst. Since the work function (Φ) of a material dictates the ease to remove an electron out of the surface, we compute the work function for one of the configuration (IV here) by considering the slab of 2 × 2 × 6.5 supercell of Cu-Te doped BTO as described in the *Computational Methodology* section. As shown in Figure 7, we have calculated the work function (defined as the potential difference between vacuum and fermi level) for both BaO and TiO_2_ terminated surfaces. We find that BaO terminated surface (Φ = 2.708 eV) has a smaller work function than the TiO_2_ terminated one (Φ = 3.554 eV) making the BaO surface easier to be oxidized by the removal of an electron from the surface.

## Conclusion

Co-doping Cu and Te atoms into tetragonal BaTiO_3_ has been explored as a route to reducing the band gap found in BaTiO_3_ while retaining its ferroelectric order. Considering a dopant concentration of 11%, we find a band gap reduction of 0.42 eV in a few dopant distribution configurations, while partially retaining the ferroelectric distortions of the host compound. In each of these cases, the nature of the band gap is also found to be an indirect one. Slab calculations with 2 × 2 × 6.5 supercell establish that the BaO terminated surface has a lower work function compared to the TiO_2_ terminated one.

## Data Availability

The original contributions presented in the study are included in the article, further inquiries can be directed to the corresponding author.
